# SAM 40: Dataset of 40 subject EEG recordings to monitor the induced-stress while performing Stroop color-word test, arithmetic task, and mirror image recognition task

**DOI:** 10.1016/j.dib.2021.107772

**Published:** 2022-01-01

**Authors:** Rajdeep Ghosh, Nabamita Deb, Kaushik Sengupta, Anurag Phukan, Nitin Choudhury, Sreshtha Kashyap, Souvik Phadikar, Ramesh Saha, Pranesh Das, Nidul Sinha, Priyanka Dutta

**Affiliations:** a Department of Information Technology, Gauhati University, Guwahati, Assam 781014, India; b Department of Electrical Engineering, National Institute of Technology, Silchar, Assam 788010, India; c Department of Computer Science and Engineering, National Institute of Technology Calicut, Kozhikode, Kerala 673601, India; d Department of Electronics and Communication Engineering, Gauhati University, Guwahati, Assam 781014, India

**Keywords:** EEG, Stroop color-word test, Short-term stress monitoring, Emotiv Epoc, Savitzky-Golay filter, Wavelet thresholding, Brain-computer interface

## Abstract

This paper presents a collection of electroencephalogram (EEG) data recorded from 40 subjects (female: 14, male: 26, mean age: 21.5 years). The dataset was recorded from the subjects while performing various tasks such as Stroop color-word test, solving arithmetic questions, identification of symmetric mirror images, and a state of relaxation. The experiment was primarily conducted to monitor the short-term stress elicited in an individual while performing the aforementioned cognitive tasks. The individual tasks were carried out for 25 s and were repeated to record three trials. The EEG was recorded using a 32-channel Emotiv Epoc Flex gel kit. The EEG data were then segmented into non-overlapping epochs of 25 s depending on the various tasks performed by the subjects. The EEG data were further processed to remove the baseline drifts by subtracting the average trend obtained using the Savitzky-Golay filter. Furthermore, the artifacts were also removed from the EEG data by applying wavelet thresholding. The dataset proposed in this paper can aid and support the research activities in the field of brain-computer interface and can also be used in the identification of patterns in the EEG data elicited due to stress.

**Specifications Table**

**Table:** 

Subject Area:	Neuroscience, Psychology.
More Specific Subject Area	Brain-Computer Interface, Experimental and Cognitive Psychology, Neuroimaging.
Type of data	32 Channel EEG time-series data
How the data were acquired	Visual stimuli corresponding to different simple cognitive tasks were presented to the healthy volunteers. The volunteers were instructed to respond according to the different tasks during which their EEG data were recorded. The visual stimuli were presented on a 24 inch monitor (Dell Inc., Texas, USA), and the EEG data were acquired with a 32-channel Emotiv Epoc Flex gel kit (Emotiv Inc., San Francisco, USA).
Data format	Raw EEG time series Processed EEG time series
Parameters for data collection	EEG was recorded from 32 channels (plus CMS/DRL references) with a dynamic range of +/- 4.12 mV, with a resolution of 0.51 µV/bit, and a range of 14 bits. EEG was sampled at 128 SPS (1024 Hz internal). Visual stimuli consisted of various images for psychologically inhibiting the cognitive interference ability of an individual.
Description of data collection	This dataset presents a collection of electroencephalographic (EEG) data recorded from 40 subjects (female: 14, male: 26, mean age: 21.5 years). The experiment was primarily conducted to monitor the short-term stress elicited in an individual while performing various tasks such as Stroop color-word test, solving arithmetic questions, identification of symmetric mirror images, and a state of relaxation. The individual tasks were carried out for 25 s and three trials were recorded for each of the individual tasks. The subjects were presented with the various stimuli on a monitor placed 70 cm away from the subjects. The subjects were further asked to give their ratings on a scale of 1–10 depending on the level of stress elicited while performing the various mental tasks (Table 1). The EEG was recorded with a 32-channel Emotiv Epoc Flex gel kit. The EEG data corresponding to the various tasks were segmented into non-overlapping epochs of 25 s. The EEG data were further processed to remove the baseline drifts by subtracting the average trend obtained using the Savitzky-Golay filter. The artifacts were also removed from the EEG data by applying wavelet thresholding.
Data source location	Department of Information Technology, Gauhati University Guwahati, India.
Data accessibility	The data are available freely and is hosted publicly. The information for the public repository is as follows: Repository name: Figshare Data identification number: https://doi.org/10.6084/m9.figshare.14562090.v1 Direct URL to data: https://doi.org/10.6084/m9.figshare.14562090.v1

**Value of the Data**

• This dataset of EEG signals is recorded to monitor the stress-induced among individuals while performing various tasks such as: performing the Stroop color-word test, solving mathematical problems, identification of symmetric mirror images, and a state of relaxation. The goal of the dataset was aimed at capturing the induced stress due to each of the individual tasks.
• This dataset will help the research communities in the identification of patterns in EEG elicited due to stress and can also be used to identify perceived stress in an individual.
• Behavioral ratings of stress levels were also collected from the participants for each of the tasks- Stroop color-word test, arithmetic problem solving, and mirror image recognition task. The ratings of the individual subjects are also provided with this dataset. These measures may prove useful for additional analysis that has not yet been explored.
• The dataset can also be utilized in clinical diagnosis for the identification of stress among subjects.
• Additional research on the source localization of the EEG signals responsible for stress can be carried out from the data. Classification of EEG signals based on cognitive tasks of an individual can be used for additional inference. Moreover, the type of task that elicits the maximum amount of stress can be analyzed from the dataset.

## 1 Data Description

This dataset is structured in two main folders (/raw_data and /filtered_data). The /raw_data folder contains the EEG time-series segmented in epochs corresponding to the experimental trials and has been named accordingly to identify the experimental events and the trial. The EEG data present in the /raw_data folder contains noise and is also corrupted with artifacts. The /filtered_data folder contains the clean EEG data and is free from different artifacts. Both raw and clean data have been provided to facilitate the research activity as different filtering methods may be applied by different researchers to remove artifacts from the EEG data. Moreover, a .xls file named scales.xls is also given along with the EEG data. The file provides the feedback by the subjects on a scale of 1–10 depending on the stress levels experienced by the subject during a particular task in a trial. Besides, a .locs file named as Coordinates.locs has also been provided to facilitate the plotting of the EEG data. Moreover, another folder named /artifact_removal is also provided which contains the code for artifact removal. The correct_EEG.m file provides the code for the artifact removal procedure implemented in Matlab. The /artifact_removal folder contains two .mat files namely Corrupted_EEG.mat and Cleaned_EEG.mat which represent a sample EEG recording before and after the removal of artifacts respectively. The two files have been provided in the folder for the purpose of demonstration of the Matlab code and have been extracted from subject 10 while performing the arithmetic task. It is to be noted that both raw EEG data and filtered EEG data have been uploaded to the portal. The /filtered data folder contains the filtered EEG data of all the segmented trials which have been obtained using the correct_EEG.m file provided in the /artifact_removal folder and the /raw_data folder contains the unfiltered raw EEG data. The artifact removal procedure has been described in the data pre-processing section. The EEG data provided in the folders are segmented according to the respective tasks- Stroop color-word test, arithmetic task, mirror image recognition task, and a state of relaxation. The corresponding files are provided in EEGLAB format and can be loaded into EEGLAB using ‘Load existing dataset’ from the ‘File’ menu. In order to visualize individual channels, ‘Channel data (scroll)’ from the ‘Plot’ menu has to be selected to generate the plot (Figs. 6 and 7 are generated using the described procedure). The description of the corresponding experimental tasks and the methodology to generate the data are described in the next section.

The past decade has witnessed an ever-increasing growth in the field of brain-computer interface (BCI). BCI's have been developed for various applications like motor imagery, prosthesis, emotion recognition, etc. Emotion plays a significant role in cognition, motivation, perception, creativity, attention, learning, and decision-making [4]. The visualization of mental states has a lot of potentials and can greatly aid psychologists in diagnosing mental disorders.

The primary goal of this dataset is to capture the level of stress elicited in individuals while performing different types of tasks such as performing the Stroop color-word test, solving arithmetic problems, and recognizing symmetric mirror images. The dataset can be used to identify the levels of stress induced in an individual, while performing different tasks.

## 2 Description of the Different Mental Tasks

The dataset is created to primarily monitor the stress induced in an individual while performing different cognitive tasks. The different cognitive tasks considered for the experiment are: the Stroop color word test, arithmetic problem solving, and recognition of symmetric images. The different tasks are described below.

### 2.1 Stroop color word test

**Fig. 1:**
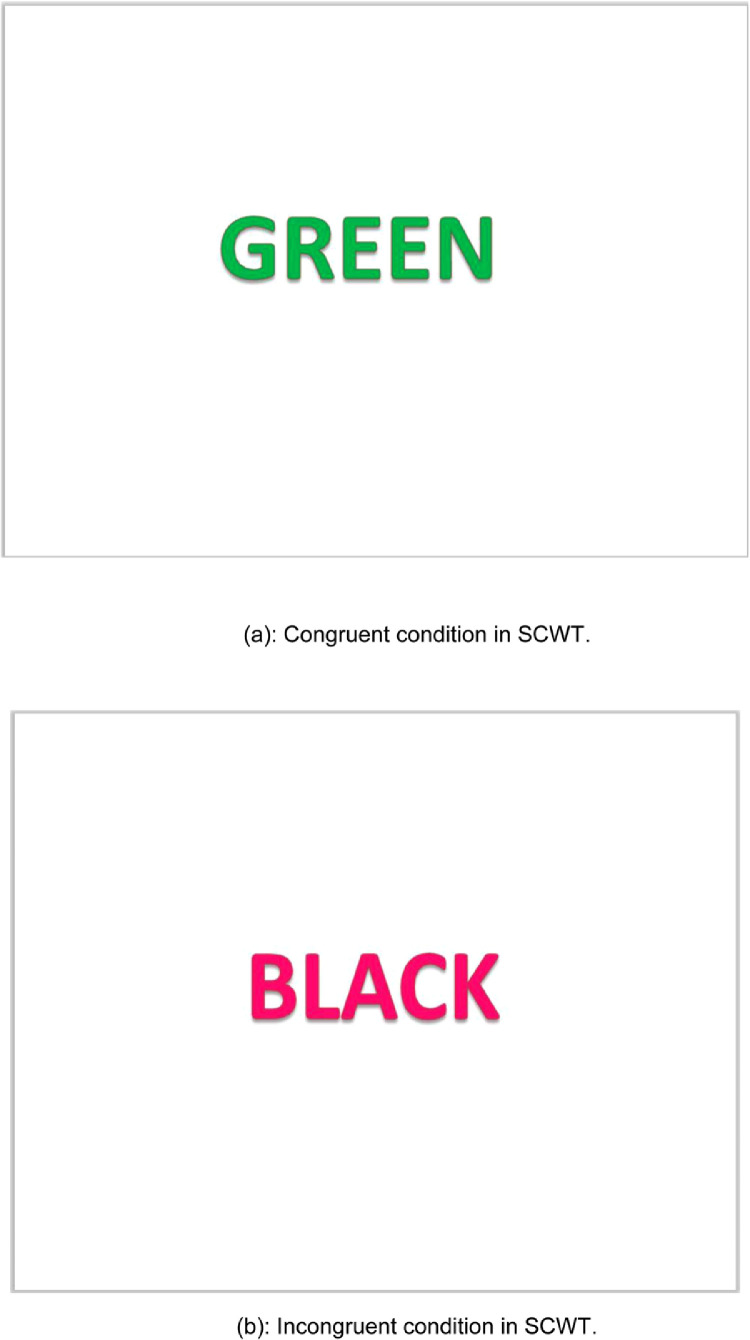
(a) Congruent condition in SCWT. (b) Incongruent condition in SCWT.

The Stroop Color- Word Test (SCWT) is a neuropsychological test used to assess the cognitive inference ability while processing multiple stimuli [1]. SCWT has been used in the literature [2] to induce stress in subjects and therefore has been adopted in the present work. The subjects are asked to identify the names of colors printed in different color patches. Accordingly, there are two conditions- congruent condition and incongruent condition. In congruent condition, the name of the color matches with the color of the ink with which the word is printed and in incongruent condition, the name of the color does not match with the ink with which the word is printed. Both the conditions are represented in Fig. 1(a) and Fig. 1(b), respectively. 11 such impulses comprising of both congruent and incongruent conditions are presented to the subject in a trial during the course of the experiment.

### 2.2 Mirror image recognition task

**Fig. 2:**
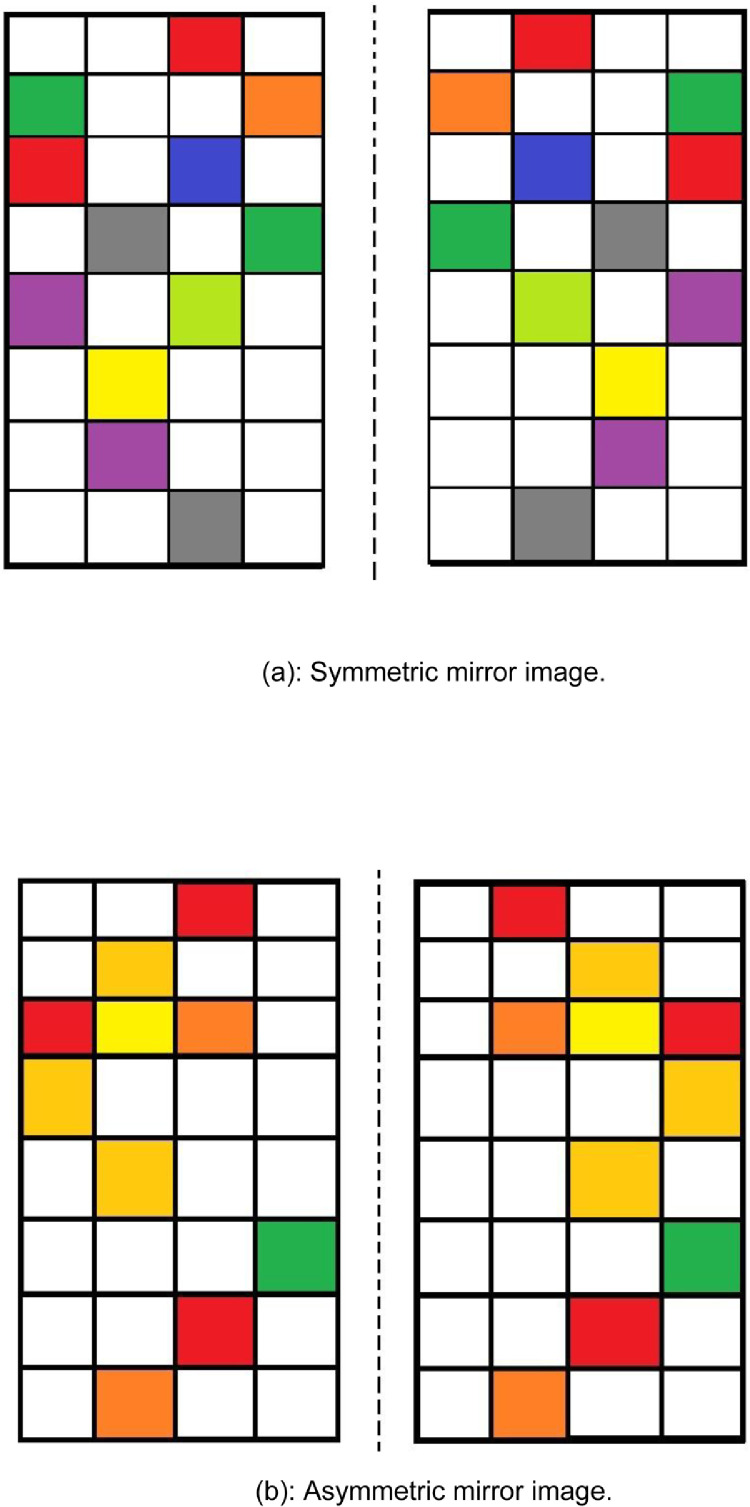
(a) Symmetric mirror image. (b) Asymmetric mirror image.

Images have also been used in the literature to induce various types of emotions [3,4] and thus have been adopted in the present work to induce stress in the subjects. In the proposed work, mirror images are presented to the subject and is asked to identify whether the displayed images are symmetric or asymmetric to each other. Fig. 2(a) and (b) shows one such symmetric and asymmetric mirror images used in the present work respectively. The mirror images have been designed so as to induce stress in students during the course of experimentation. 8 such images have been used in the proposed work for eliciting stress in individuals during a trial.

### 2.3 Arithmetic problem solving task

**Fig. 3:**
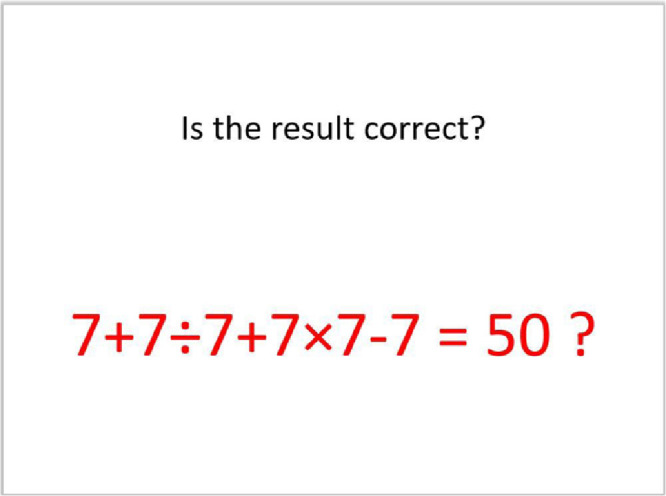
An Arithmetic task stimuli.

Arithmetic problem solving task is known to elicit stress in individuals [5]. In the proposed work, the subject is asked to mentally solve the problem and respond with a thumbs up or thumbs down gesture depending on whether the answer displayed on the screen is a correct solution for the arithmetic problem or not. Fig. 3 depicts one such arithmetic stimuli used in the proposed work. 6 such arithmetic stimuli involving different arithmetic operators are presented in a trial to the subject during the course of the experiment.

## 3 Experimental Design, Materials and Methods

**Fig. 4:**
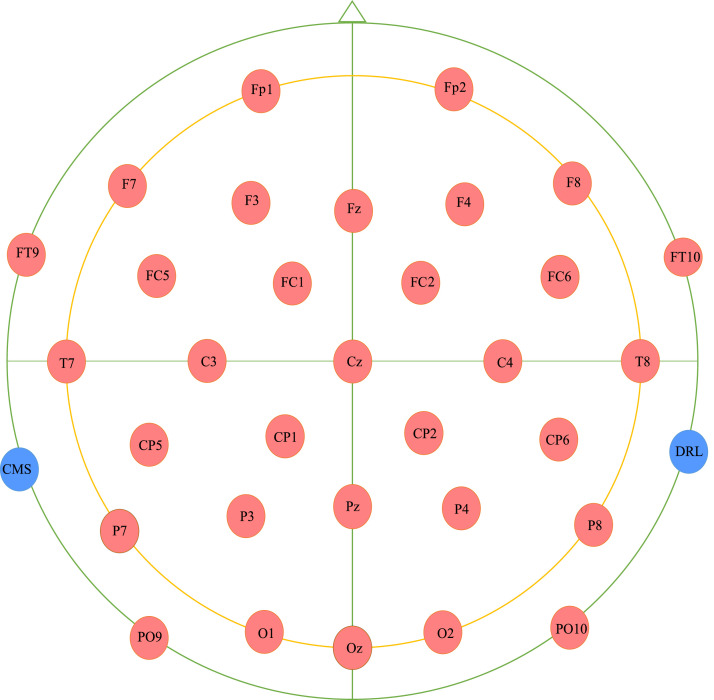
Placement of the 32 electrodes.

The data were collected primarily from the students studying in the institute. 14 female and 26 male students participated in the experiment. The age of the subjects ranged from 18 to 25 years with a mean age of 21.5 years. The subjects were asked to solve the tasks within a specified time. Written consent was obtained from the individual subject before participating in the experiment. EEG data were recorded from 32-channels using Emotiv Epoc Flex gel kit at a sampling frequency of 128 Hz. The channels considered for recording the brain activity were- C_Z_, F_Z_, Fp_1_, F_7_, F_3_, FC_1_, C_3_, FC_5_, FT_9_, T_7_, CP_5_, CP_1_, P_3_, P_7_, PO_9_, O_1_, P_Z_, O_Z_, O_2_, PO_10_, P_8_, P_4_, CP_2_, CP_6_, T_8_, FT_10_, FC_6_, C_4_, FC_2_, F_4_, F_8_, and, Fp_2_. Fig. 4 shows the placement of the different electrodes on the head of an individual. CMS and DRL are two reference electrodes connected to the left and the right mastoid region of the head respectively. Three trials were recorded for an individual subject. The trail recording paradigm is described in the next section.

### 3.1 Data recording methodology

The experiment is set up by the experimenters in the beginning. Then the EEG device is mounted over the subject and instructions are given to the subject regarding the experiment. Then the experimenter starts to record the EEG data and the subject is asked to perform the various tasks. The subject is initially asked to relax for 25 s where relaxing music is played to ease the subject. After which, the instructions for the Stroop color-word test is shown to the subjects. The subject is asked to perform the Stroop color-word test for 25 s. The subject then relaxes for 5 s and then the instructions for the next task are displayed for 10 s. In the next phase, the subject is shown different mirror images and is asked to identify whether the images are symmetric or asymmetric and respond with a thumbs up or thumbs down gesture depending on whether the images displayed represent symmetric mirror images or not. The mirror image symmetry task is carried out for 25 s, after which the subject again relaxes for 5 s and then the instructions for the next task are displayed for 10 s. Finally, the subject is instructed to solve arithmetic problems where the subject is asked to mentally solve the problem and respond with a thumbs up or thumbs down gesture depending on whether the answer displayed on the screen is a correct solution for the corresponding arithmetic problem or not. The arithmetic task is also carried out for 25 s. The completion of the arithmetic task marks the completion of a trial. Moreover, when the subject is responding, an operator also gives feedback as to whether the answers provided by the subject are incorrect or correct.

After finishing an individual trial, the subject is asked to rate the tasks on a scale of 1–10 depending on the level of stress experienced during the particular tasks. A rating of 10 on the scale represents a high level of stress being induced on the subjects and a rating of 1 representing the minimal amount of stress being experienced by the subjects.

**Fig. 5:**
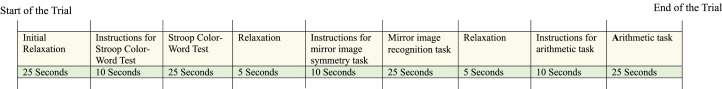
Trial recording paradigm.

After collecting the responses from the subject, the next trial is recorded. The next trial is repeated in the same order, but with a different set of questions as the subject might become familiarized with the questions. 3 trials were recorded from an individual subject. Fig. 5 represents the trail recording paradigm followed in the experiment and Table 1 lists the individual ratings for different tasks in a trial given by a specific subject.

**Table 1:** Tabular representation of verbal feedback from the observer after each trial on a scale of 1–10.

	Trial_1			Trial_2			Trial_3		
Subject No.	Stroop Color-word Test	Arithmetic Task	Mirror Image Recognition Task	Stroop Color-word Test	Arithmetic Task	Mirror Image Recognition Task	Stroop Color-word Test	Arithmetic Task	Mirror Image Recognition Task
**1**	3	6	3	2	7	5	4	4	7
**2**	5	3	4	4	3	4	3	7	5
**3**	4	5	3	5	3	5	5	8	7
**4**	4	5	3	2	3	5	5	7	5
**5**	6	6	6	2	5	3	3	5	7
**6**	2	5	4	3	4	3	5	8	6
**7**	4	5	5	3	3	3	6	6	3
**8**	4	3	5	3	6	6	3	5	6
**9**	4	3	6	4	4	4	6	7	4
**10**	4	3	4	3	4	5	5	6	4
**11**	4	7	3	5	6	5	5	5	6
**12**	2	5	7	5	5	4	3	8	6
**13**	3	3	5	2	3	4	1	3	5
**14**	3	3	4	2	6	4	4	6	3
**15**	5	9	6	3	7	5	5	6	5
**16**	1	4	4	1	5	6	6	8	5
**17**	8	8	9	4	6	8	6	7	7
**18**	6	6	5	5	5	6	7	9	6
**19**	6	4	6	3	2	4	3	4	1
**20**	7	10	9	5	10	9	6	10	8
**21**	9	7	8	9	8	8	9	7	8
**22**	5	9	8	6	8	8	5	7	6
**23**	1	3	2	1	4	2	5	8	4
**24**	1	4	2	1	2	1	4	5	7
**25**	2	2	1	2	2	1	5	5	7
**26**	8	6	7	7	6	8	6	4	5
**27**	3	5	5	2	3	4	3	5	3
**28**	7	7	8	5	7	7	4	6	5
**29**	6	6	7	6	8	7	5	6	6
**30**	5	7	6	5	8	6	4	7	5
**31**	4	10	8	3	9	7	7	6	3
**32**	1	5	5	1	1	2	3	7	6
**33**	3	5	4	2	3	2	6	8	3
**34**	1	2	3	1	3	1	3	2	2
**35**	1	6	5	1	5	2	5	4	6
**36**	6	4	4	3	5	4	5	5	5
**37**	4	6	5	2	5	3	6	8	4
**38**	5	6	4	3	5	6	3	4	5
**39**	3	6	4	3	5	5	5	6	3
**40**	5	3	5	5	4	6	3	4	4

### 3.2 Data pre-processing

**Fig. 6:**
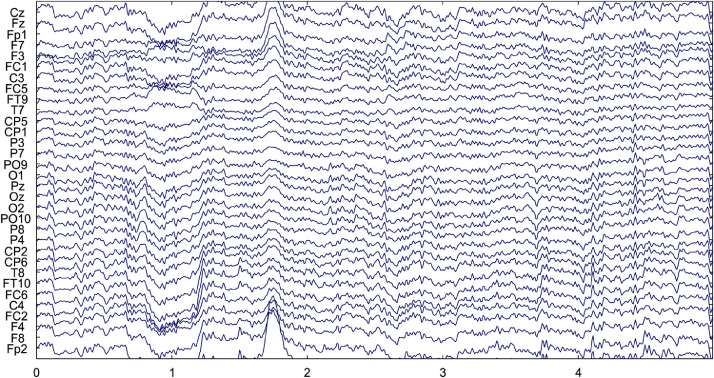
EEG Data of Subject 10 before artifact removal.

The raw data was imported and clipped in Matlab R2019a. Band-pass filtering in the range of 0.5–45 Hz was applied to the data initially. The collected data were contaminated with different types of artifacts. Fig. 6 represents one such plot of EEG data before artifact removal for subject 10 while performing the arithmetic task in the third trial. The figure has been generated using the EEGLAB toolbox as described in the data description section. It is evident from Fig. 6 that the EEG data is corrupted with different types of artifacts.

Components containing artifacts (i.e., eye movements, eye blinks, muscular activity, etc.) were identified and removed using a combination of Savitzky-Golay filter and wavelet thresholding [6,7]. Artifacts are signals caused by muscle movements and eye movements which corrupt the original EEG signal. Savitzky-Golay smoothing filters are used to "smooth out" a noisy signal. The Savitzky-Golay filter is created with a frame length of 127 and an order of 5. The Savitzky-Golay smoothing filters are used to create a reference signal, which is subtracted from the EEG data to remove the average trend in the EEG data. After removing the average trend from the EEG signal, wavelet thresholding is applied to remove the components which have amplitude values over a certain threshold in different scales. The signal is decomposed up to 4 levels with ‘db2’ (Daubechies 2) as the mother wavelet. A threshold of 0.8 times the standard deviation of the detailed coefficient at the third level of decomposition is selected for thresholding. The thresholding removes the remaining components which were not removed after subtraction of the average trend from the EEG. The artifact removal procedure has been given in the correct_EEG.m file within the /artifact_removal folder.

Both Raw EEG data and filtered EEG data have been uploaded to facilitate research as different artifact removal methods can be applied by different researchers on the EEG data which can make the analysis more efficient.

**Fig. 7:**
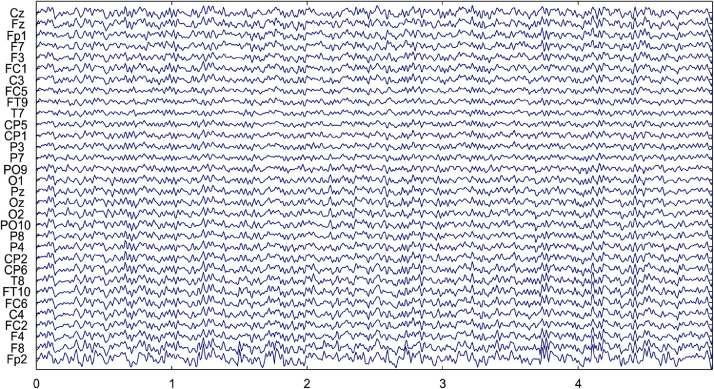
EEG Data of Subject 10 after artifact removal.

Fig. 7 represents the corrected version of the EEG represented in Fig. 6. It can be observed from Fig. 7 that the artifact removal procedure adopted in the proposed work efficiently removes the artifacts from the EEG data and preserves good correlation in the EEG data.

### 3.3 Naming convention

**Fig. 8:**
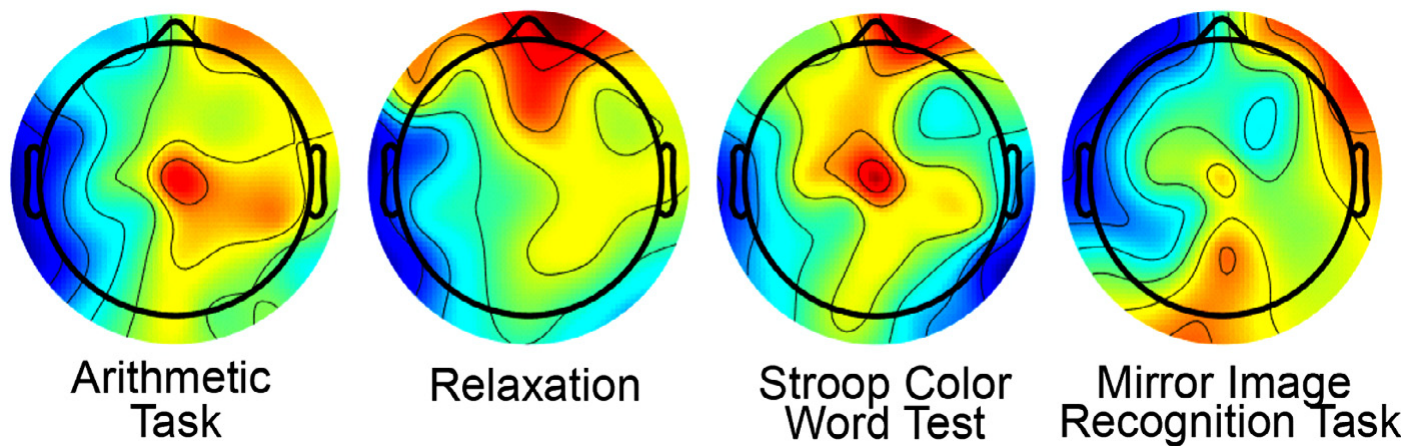
EEG topography plot from clean data.

EEG data files contain four types of event codes: (i) Mat files that correspond to the relaxation phase are marked as ‘relax’. Please note that the files also mark the subject and the trials. (ii) Mat files that correspond to the Stroop color-word test are marked as ‘Stroop’ (iii) Mat files that correspond to the mirror image recognition task are marked as ‘Mirror_image’ and (iv) Mat files that correspond to the arithmetic problems are marked as ‘Arithmetic’. Fig. 8 represents the topographic plot of the C_Z_ electrode of subject 17 w.r.t the different tasks performed by the subject. The plots have been generated by using the ‘topoplot’ function available in the EEGLAB toolbox.

### 3.4 Observer's feedback

After each trial, the subject's feedback in terms of the level of stress experienced during different tasks is taken on a scale of 1 to 10. The ratings have been taken to correlate the EEG data to the amount of stress experienced by the subjects. A rating of 10 on the scale represents a high level of stress getting induced on the subjects and a rating of 1 represents the minimal amount of stress getting induced on the subjects.

## Ethics Statement

Ethical approval was obtained from the Institutional Ethics Committee, Gauhati University, reference no. GUIEC/2019/019 dated: 15/10/2019. The experiment involved human subjects in research whose participation was completely consensual, anonymous, and voluntary. Before opting to partake in the study, the participants were informed about the nature of the study. The data collection was conducted according to the Declaration of Helsinki.

## Consent from Participants

Informed consent was obtained from all the subjects participating in the study.

## CRediT authorship contribution statement

**Rajdeep Ghosh:** Conceptualization, Methodology, Data curation, Project administration. **Nabamita Deb:** Conceptualization, Supervision, Writing – original draft. **Kaushik Sengupta:** Investigation, Software, Validation. **Anurag Phukan:** Investigation, Writing – review & editing. **Nitin Choudhury:** Software, Data curation. **Sreshtha Kashyap:** Visualization. **Souvik Phadikar:** Formal analysis. **Ramesh Saha:** Writing – review & editing. **Pranesh Das:** Conceptualization. **Nidul Sinha:** Conceptualization, Methodology. **Priyanka Dutta:** Visualization.

## Declaration of Competing Interest

The authors declare that there is no known competing financial interests or personal relationships which have, or could have influenced the work reported in this article.
